# Early diagnosis of sepsis using an E-health application for a clinical early warning system outside of the intensive care unit: a case report

**DOI:** 10.1186/s13256-022-03385-9

**Published:** 2022-05-09

**Authors:** Daniel Aiham Ghazali, Philippe Kenway, Christophe Choquet, Enrique Casalino

**Affiliations:** 1 Emergency Department and Emergency Medical Service, Bichat University Hospital, AP-HP, 46 rue Huchard, 75018 Paris, France; 2 Emergency Department, Amiens University Hospital, Amiens, France; 3 IAME (Infection, Antimicrobial, Modeling, Evaluation), UMR 1137 INSERM, University of Paris, Paris, France

**Keywords:** E-health, Older patient, Sepsis, Emergency medicine, Intensive care unit

## Abstract

**Background:**

Elderly and frail patients who are unable to call for help in case of vital distress can develop complications during their hospitalization. As a supplement to clinical monitoring by the nursing staff, these patients can also be monitored in real time, with the Sensium E-health technology. An application notifies clinical staff of any change in their vital signs (heart rate, respiratory rate, temperature) outside of normal ranges, suggestive of physiological decline. Nurses and physicians are notified of these abnormal changes by email and also via mobile application (iPhone or iPad), allowing early intervention to prevent further deterioration.

**Case presentation:**

An 86-year-old Caucasian female, with chronic kidney disease, was hospitalized in our medical unit for pyelonephritis associated with a moderate deterioration of serum creatinine. Remote continuous monitoring allowed us to diagnose clinical deterioration early and adjust her treatment. The treatment improved her clinical condition and amended the secondary sepsis with circulation failure in 2 days.

**Conclusions:**

The prognosis for patients with acute complicated pyelonephritis is much worse than for those with uncomplicated pyelonephritis. Remote continuous monitoring might be helpful to early diagnose urosepsis. This technology leads to improved prognosis of patients without initial vital distress, allowing early treatment and admission to intensive care unit.

## Background

Older and frail patients having an infectious disease without vital distress are hospitalized outside of an intensive care unit (ICU). These patients have a major risk of deterioration in the first hours after their treatment is initiated. Moreover, in case of hemodynamic or respiratory worsening related to sepsis, these patients are usually unable to call for help [1]. E-health technology can assist nurses and physicians in the management of patients when hospitalization in the ICU is not initially required [2]. In hospital wards, where the nurse-to-patient ratio is lower than in post-anesthesia care units or ICUs, one-third of vital sign spot checks are not done on time [3]. Medical-grade wireless and wearable sensors facilitate continuous monitoring and may improve outcomes in hospital wards [4]. However, most studies to date have explored the use of remote monitoring for cardiovascular diseases or surgical and postoperative complications [5]. The present case report illustrates the benefit of using remote continuous monitoring for the early detection of sepsis outside the ICU, revealed by increase in heart rate (HR) and respiration rate (RR). In our hospital, the remote monitoring technology deployed is the Sensium E-health system, which measures vital parameters via wireless connection every two minutes, thus enabling the closer monitoring of ward-based patients [6]. Nurses and physicians are alerted by email and with the iOS application (iPhone or iPad, Apple) in case of any change of the patients’ vital signs (HR, RR, temperature) and allows the calculation of early warning scores.

## Case presentation

**Fig. 1:**
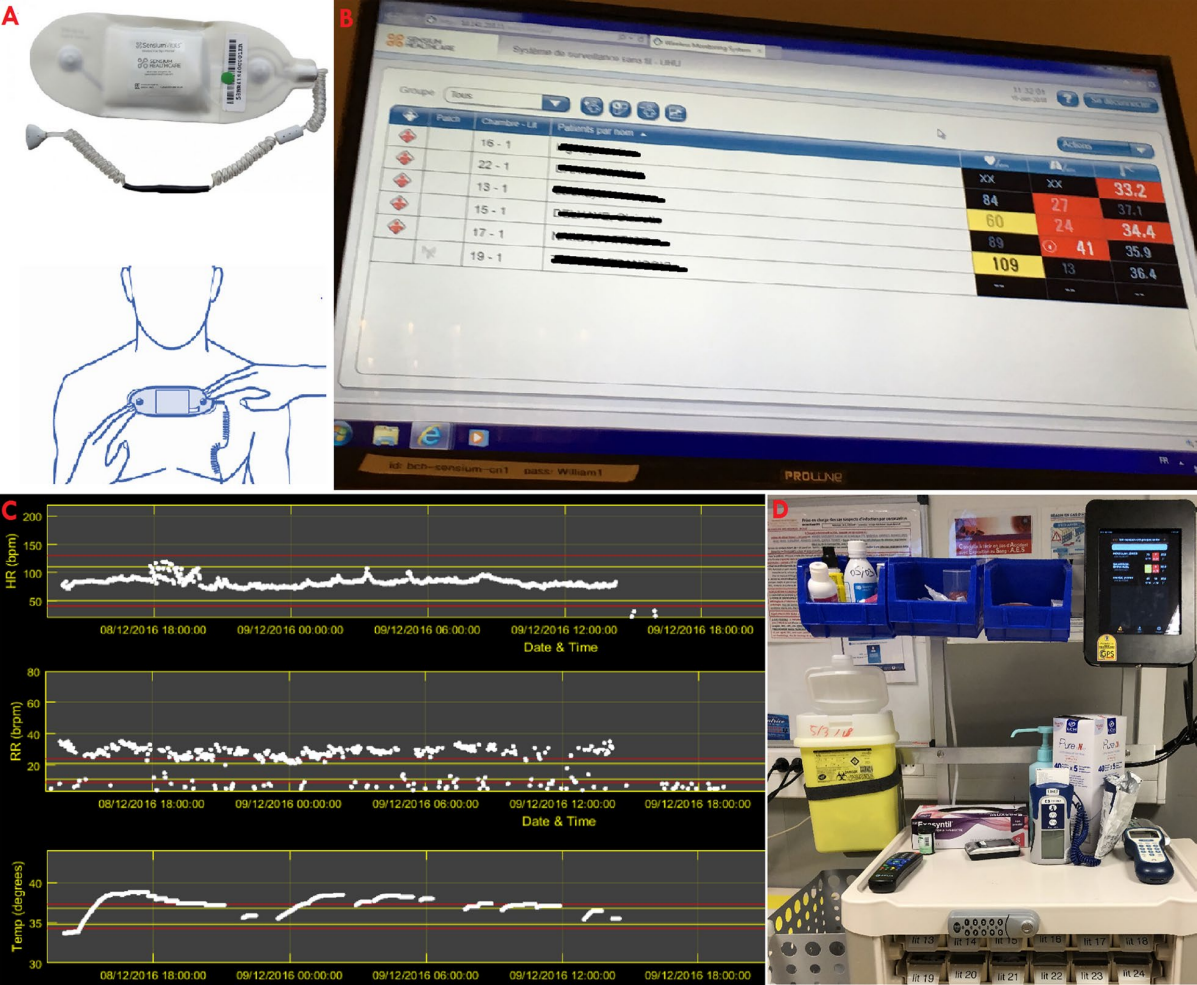
Clinical early warning system. **A** Wireless monitoring system. **B** Sensium application for continuous monitoring of vital signs. **C** Software with analysis of vital signs. **D** iPad fixed on the nurse trolley for continuous monitoring of vital signs

**Fig. 2:**
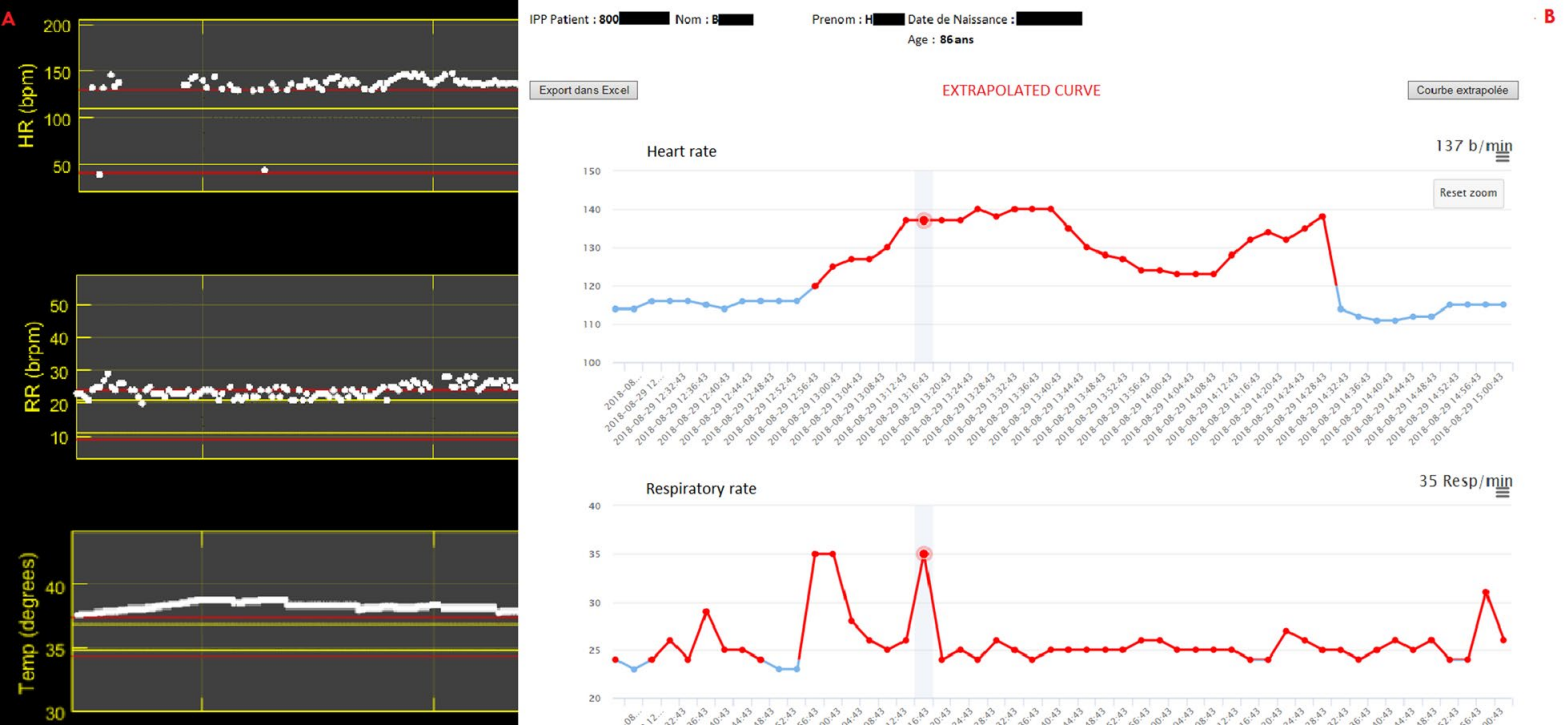
Early diagnosis of severe sepsis using Sensium E-health technology. **A** Wireless monitoring system with analysis of vital signs. **B** Extrapolated curves; HR, heart rate; RR, respiration rate; Temp, temperature

**Fig. 3:**
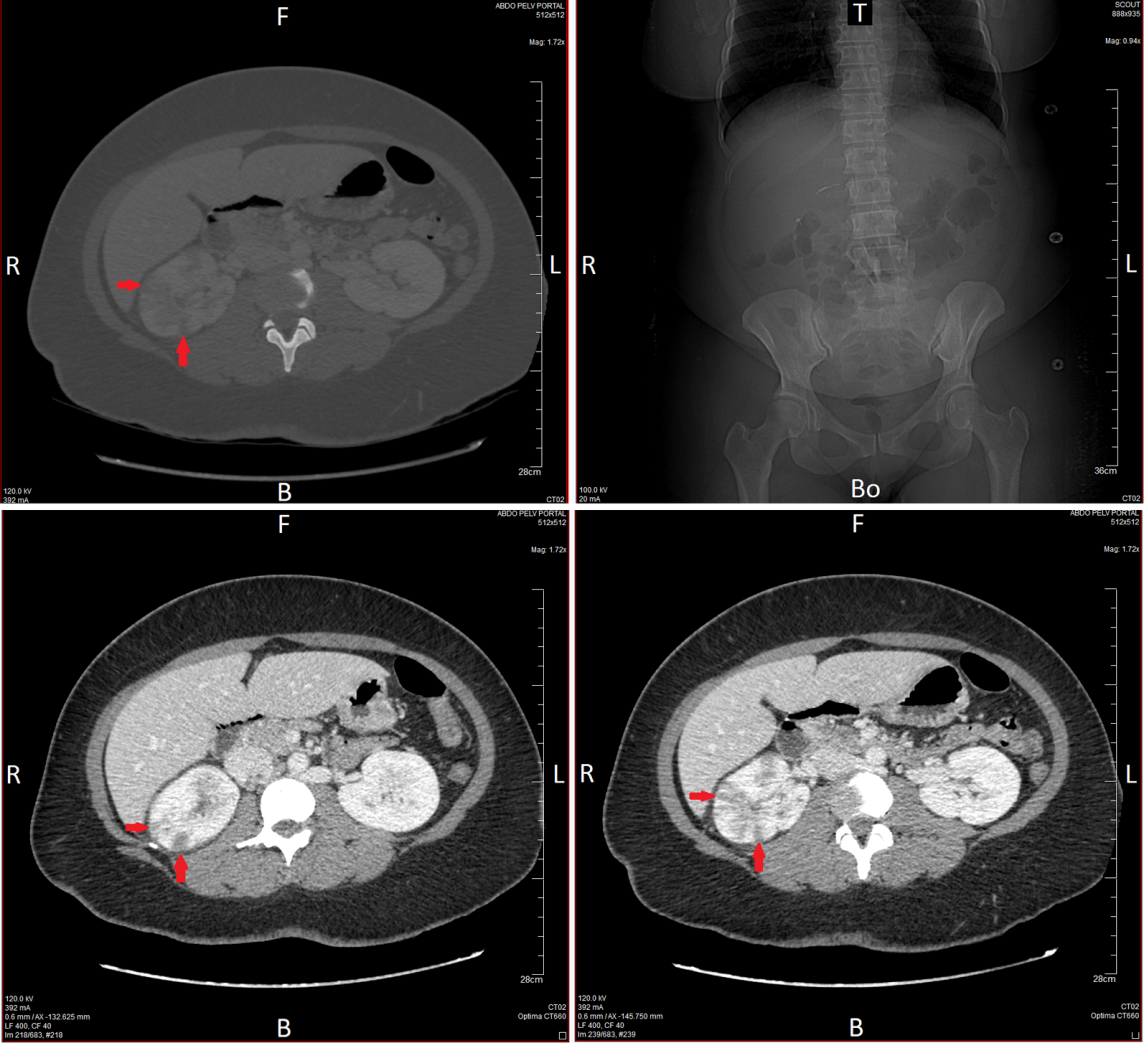
Multiple foci of nephritis of the upper pole of the right kidney. Axial CT slice through the abdomen from CT scans without injection of intravenous contrast (photo 1), CT Scout view (photo 2), and with injection of intravenous contrast (photos 3 and 4). B, back; Bo, bottom; F, front; L, left; R, right; T, top. CT scan found multiple triangular hypodensities (indicated by the red arrows) of the right superior polar renal cortex in favor of foci of nephritis. Absence of contrast of the right pyelic walls. There was no dilatation of the right and left pyelocaliceal cavities. There was no parenchymal abnormality of the left renal pelvis. The bladder was in low replenishment. There was no evidence of lithiasis in the urinary excretory tract. There was no abnormality in the hepatic, splenic, pancreatic, or adrenal compartments. No intraperitoneal or retroperitoneal effusion was observed. There was a good permeability of the vascular axes. Conclusion: multiple foci of nephritis of the upper pole of the right kidney; no dilatation of the pyelocaliceal cavities or obstruction of the urinary excretory tract

An 86-year-old Caucasian female had a history of high blood pressure and diabetes with chronic kidney disease. Gynecologic and obstetric history were gravida 2, para 2 (G2P2), with two healthy children. The onset of menopause was at the age of 51 years. She was a widow, lived alone, and was a former schoolteacher retired since the age of 65 years. She did not smoke or drink alcohol. Her medications were: perindopril (oral route) 4 mg once a day, metformin (oral route) 500 mg two times a day, and insulin glargine injection 100 units/mL, 16 IU once a day at the same time. She was hospitalized in our medical unit within the emergency department for pyelonephritis associated with a moderate deterioration of the serum creatinine from 114 μmol/L to 139 μmol/L (normal range 50–100 μmol/L). Her initial vital signs included RR of 19 breaths per minute, HR of 112 beats per minute with regular pulse, and blood pressure (BP) of 107/59 mmHg with mean arterial pressure (MAP) of 75 mmHg. Initial clinical examination of this patient revealed that she had a temperature of 38.9 °C with sweating, unilateral left flank pain, and nausea. Cardiovascular, pulmonary, and neurological examinations were normal. Laboratory investigations indicated a bacterial infection with procalcitonin of 0.56 µg/L and white blood cell count of 24.0 × 10^3^/mm^3^ (normal range 4–10 × 10^3^/mm^3^) including 21.6 × 10^3^/mm^3^ neutrophils. Initial arterial lactate was 1.90 mmol/L. Other blood tests were within the reference range (Table 1). Urine dipstick test confirmed a urinary tract infection with positive dipstick hematuria, and leukocyte esterase and nitrite tests returned positive. The treatment consisted of intravenous administration of cefotaxime 1 g/8 hours antibiotic and 0.9% saline 500 mL over a period of 30 minutes and then 1000 mL/12 hours, and pain and fever management. HR was 91 beats per minute, blood pressure 135/79 mmHg (MAP 98 mmHg), and serum lactate 1.3 mmol/L after treatment. Remote continuous monitoring was used in the ward to monitor the patient in real time (Figure 1A) in addition to the nursing monitoring, which included the measurement of vital signs every 8 hours. Remote monitoring does not replace nursing monitoring, which also records the assessment of pain and other patient complaints as well as delivering care. By using a patch worn on the patient chest associated with an axillary temperature sensor, smart algorithms continuously process and analyze vital signs [6, 7]. The E-health technology aimed to generate targeted notifications of patient deterioration. The objective was to detect a possible deterioration of the vital signs in the time periods between the manual monitoring by the nurses in the hospital setting. In addition to regular monitoring on standard Personal Computer (PC) stations (Fig. 1B and C), surveillance was also conducted by iPad (Apple). iPads were attached to trolleys used by nurses, allowing them to continually monitor all patients while doing rounds (Fig. 1D). In the present case, the emergency nurse and physician received an alert by email and the Sensium application on 29 August 2018 at 12:56 pm indicating a sudden increase of HR from 115 to 140 beats per minute, an increased RR from 22 to 35 breaths per minute, and an elevated temperature of 39.6 °C (Fig. 2A and B). Computed tomography (CT) scan was carried out and found multiple foci of nephritis of the upper pole of the right kidney. No dilatation of the pyelocaliceal cavities or obstruction of the urinary excretory tract was seen (Fig. 3). Upon manual monitoring, clinical examination, and biological tests, sepsis was diagnosed on the basis of the Sequential [Sepsis-related] Organ Failure Assessment (SOFA) score calculation [8]. SOFA was 6: Coma Glasgow Score (CGS) 14, BP 97/48 mmHg (MAP 64 mmHg), Arterial partial pressure of oxygen (PaO_2_) 76 mmHg and PaO_2_/The fraction of inspired oxygen (FiO_2_) 362, deterioration of the serum creatinine from 139 to 172 μmol/L, and platelet count 141,000/µL. There was no bilirubin abnormality. Therapeutic reinforcement was performed with oxygen therapy 2 L per minute because PaO_2_/FiO_2_ ratio was < 400; a single daily dose of amikacin 30 mg/kg (1800 mg intravenous injection over a period of 30 minutes) in addition to cefotaxime 2 g intravenous injection, 1 L of isotonic crystalloid 0.9% saline in 15 minutes because MAP was < 70 mmHg; and paracetamol 1 g intravenously. This treatment improved her clinical condition and reversed vital distress at 02:32 pm with HR of 112 beats per minute and RR of 22 breaths per minute (Fig. 2B and C). She was admitted to the intensive care unit. Blood culture and cytobacteriological examination of urine found *Escherichia coli* [10^6^ colony-forming units (CFU)/mL] with established sensitivity to third-generation broad-spectrum bactericidal cephalosporin antibiotics (Table 2). Treatment with cefotaxime 6 g per day was continued at a dosage of 2 g/8 hours intravenously. Her vital signs stabilized within 2 days, and she returned to a lower acute ward: HR 90 beats per minute, RR 17 breaths per minute, Glasgow Coma Score (GCS) 15, MAP 87 mmHg, PaO_2_ 91 mmHg, serum creatinine 116 μmol/L, and platelet count 183,000/µL. Antibiotic was changed at the fifth day for ciprofloxacin twice a day (every 12 hours) in the morning and evening, based on the antibiogram (Table 2). After 8 days of hospitalization, the patient was allowed to return home with treatment by ciprofloxacin twice a day (every 12 hours) in the morning and evening for a duration of 7 days. A follow-up of the patient was carried out at 7 days, which did not show any anomaly. No complications were observed by her general practitioner at 1 month, 6 months, and 1 year.

**Table 1:** Initial blood sample in the emergency department

Examination	Result	Unit	Usual value	Interpretation
Blood chemistry tests				
Glucose	8.4	mmol/L	4.1–5.9	Above high normal
Sodium	137	mmol/L	135–145	Normal
Potassium	4.4	mmol/L	3.5–5	Normal
Chlorine	98	mmol/L	98–108	Normal
Bicarbonate	27	mmol/L	24–32	Normal
Calcium	2.58	mmol/L	2.18–2.6	Normal
Phosphorus	1.38	mmol/L	0.78–1.45	Normal
Anion gap	16	mmol/L	–	
Plasma proteins	74	g/L	57–82	Normal
Creatinine	139	µmol/L	44–71	Above high normal
Urea	8.7	mmol/L	3.2–8.2	Above high normal
Creatinine clearance (MDRD equation)	33	mL/min	> 90	Below low normal
Aspartate aminotransferase (AST)	96	U/L	13–40	Above high normal
Alanine aminotransferase (ALT)	122	U/L	< 40	Above high normal
Total bilirubin	42	µmol/L	5–21	Above high normal
Indirect bilirubin	21	µmol/L	< 18	Above high normal
Alkaline phosphatase	217	U/L	46–116	Above high normal
Gamma-glutamyl transferase	203	U/L	< 38	Above high normal
Lipase	24	U/L	12–53	Normal
CRP (C-reactive protein)	199.4	mg/L	< 5	Above high normal
Procalcitonin	0.56	µg/L		
Complete blood count (CBC)				
White blood cells	24.0	10^3^/mm^3^	4–10	Above high normal
Red blood cells	4.43	10^6^/mm^3^	3.8–5.2	Normal
Hemoglobin	14.9	g/dL	11.5–16	Normal
Hematocrit	40.8	%	35–45	Normal
Mean blood volume	92.1	µL^3^	80–100	Normal
Mean blood content	30.0	pg	27–32	Normal
Blood Hb concentration	32.6	%	32–36.5	Normal
Platelets	195	10^3^/mm^3^	150–400	Normal
Formula: neutrophils	90.1%, 21.6 × 10^3^/mm^3^	–	–	
Formula: eosinophilic polynuclears	0.0%, 0.0 × 10^3^/mm^3^	–	–	
Formula: basophilic polynuclears	0.2%, 0.0 × 10^3^/mm^3^	–	–	
Formula: lymphocytes	3.8%, 0.9 × 10^3^/mm^3^	–	–	
Formula: monocytes	5.9%, 1.4 × 10^3^/mm^3^	–	–	
Blood clotting tests				
PT: control	11.7	Seconds	–	
PT: patient	13.1	Seconds	–	
PT: prothrombin rate	80	%	70–100	Normal
INR	1.11	–	–	
aPTT (control)	24.4	Seconds	–	
aPTT (sick)	23.6	Seconds	–	
aPTT (ratio)	0.97	Null	≤ 1.2	Normal
Arterial blood gas				
pH	7.426	Null	7.38–7.42	Above high normal
PaO_2_	76.0	mmHg	80–100	Below low normal
PaCO_2_	39.2	mmHg	38–42	Normal
HCO_3_	26.5	mmol/L	21–28	Normal
Base excess/deficit	1.4	mmol/L	–	
SaO_2_	94.0	%	95–99	Below low normal
Arterial lactates	1.90	mmol/L	< 1.6	Above high normal

*PT* prothrombin, *INR* International Normalized Ratio, *aPTT* Activated Partial Thromboplastin Time, *pH* potential of hydrogen, *SaO*_2_ oxygen saturation, *PaO*_2_ partial pressure of oxygen, *PaCO*_2_ partial pressure of carbon dioxide, *HCO*_3_ Concentration of hydrogen carbonate, *Hb* Hemoglobin, *MDRD* Modification of Diet in Renal Disease

**Table 2:** Urine culture and antimicrobial susceptibility test results

Examination	Result	Unit	Usual value
Direct examination			
Number of red blood cells	6	10^3^/mL	≤ 10.10^3^/mL
Number of leukocytes	126	10^3^/mL	≤ 10.10^3^/mL
Semi-quantitative cytology			
Epithelial cells	Some		
Oxalate crystals	Some		
Culture			
Bacterial species: *Escherichia coli*	10^6^	CFU/mL	
Antibiotic susceptibility testing *Dilution method in liquid medium (Vitek 2 Biomérieux)*			
Bacterial species: *Escherichia coli*			
Ampicillin	R		
Amoxicillin + clavulanic acid	R		
Ticarcillin	R		
Piperacillin + tazobactam	S		
Mecillinam	S		
Cefoxitine	S		
Cefixime	S		
Ceftazidim	S		
Ceftriaxone	S		
Amikacin	S		
Gentamicin	S		
Ofloxacin	S		
Fosfomycin	S		
Nitrofurantoin	S		
Trimethoprim + sulfonamides	S		

*R* resistance, *S* sensitivity

## Discussion

This case presentation reported a case of right acute pyelonephritis due to *Escherichia coli* leading to urosepsis. Because of the patient’s high-risk condition with chronic kidney disease and deteriorating renal function, as well as the initial fluid repletion with isotonic crystalloid solution, the patient was hospitalized. In the absence of initial vital distress, she was transferred from the ICU to a medical ward with real-time monitoring using E-health technology in addition to the usual clinical monitoring by the nurse staff, which was carried out three times per day. The E-health technology revealed the first signs of deterioration before manual vital observations and led to treatment of urosepsis quicker than would have been the case if a nurse had performed clinical surveillance. The evolution toward complicated pyelonephritis led us to perform a blood culture and an ultrasound, then a second CT scan, in addition to the urine culture in accordance with the recommendations [9]. The early diagnosis of sepsis led to an adaptation of the treatment with, among others, the addition of amikacin in accordance with the recommendations [9]. Early diagnosis and treatment reduced complication of shock, reducing the length of stay in the hospital and avoiding complications at 1 year.

Urinary tract infection is the most common bacterial infection and mainly caused by *Escherichia coli*. Sepsis is one of the complications of acute pyelonephritis and is the main factor influencing the prognosis of pyelonephritis [10]. Urosepsis has a mortality of 20–40%, and as the population ages, the incidence rise [11]. Sepsis is defined as life-threatening organ dysfunction caused by a dysregulated host response to infection. Clinically, organ dysfunction is indicated by an increase in the SOFA score of 2 points or more [12]. Early diagnosis of sepsis and septic shock enables early treatment, leading to clinical improvement [13]. This case report highlights that wireless and wearable sensors facilitate continuous monitoring and may improve outcomes in hospital wards. In septic pathologies, these systems have been described in the context of pulmonary sepsis [4] but not in sepsis of other origins. In the present case, the patient was diagnosed with pyelonephritis, based on the combination of the clinical symptoms and a positive urine dipstick test. The hospitalization was indicated because the patient was over 60 years old and presented with persistent vomiting [14]. The patient did not initially require management in ICU, since there was initially no sign of sepsis and serum lactate level was less than 2 mmol/L [15]. However, the interval between each round of clinical surveillance by a nurse, usually 6–8 hours, can delay the observation of clinical deterioration if the patient is unable to call caregivers for help. Because of initial tachycardia and abnormal renal function, this patient was monitored in real time, using remote continuous monitoring in our medical unit. This clinical early warning system allowed faster treatment after evidence of sepsis, as previously suggested by Downey *et al*. [2]. Conversely, it is not possible to admit these patients at high risk of complications without signs of vital distress to an ICU in anticipation of potential deterioration. Early appropriate antibiotic administration reduces in-hospital mortality [16]. Adjuvant treatment with oxygen therapy and early aggressive fluid therapy should be beneficial in sepsis resuscitation [17]. The two earliest indicators of patient deterioration are RR and HR [18, 19]. This wireless system is a way of deciding whether to admit a patient to an ICU according to clinical evolution after early diagnosis and treatment. It is not a substitute for a reference monitoring in ICU but can be useful for decision-making. It may provide a benefit in terms of improved patient outcomes and cost efficiency [6]. To make the deployment of the system cost-effective, in addition to using each patch to monitor a patient over several days to lower the daily cost (each single-use patch has a 5-day lifetime), it is possible to target patients for whom the risk of complications is non-negligible. Thus, this clinical case highlights the possibility of using this innovative system to improve the management of older and frail patients with an initial isolated RR or HR abnormality without any other associated failure that would justify admission to ICU. The continuous monitoring of vital signs outside the ICU is feasible using smart technology (laptop, iPhone, or iPad) integrated into the nurses’ trolleys to improve nursing monitoring. Several wearables and wireless sensors were developed to detect patient deterioration early. Breteler *et al*. compared some of these systems with an ICU monitoring system [20]. All of them were highly accurate for HR. For RR, the accuracies of the Massimo Radius-7 (Masimo Corporation, USA), EarlySense (EarlySense Ltd., Israel), and SensiumVitals (Sensium Healthcare Ltd., United Kingdom) were within a predefined acceptable range [20].

## Conclusion

Urinary tract infection, the most common bacterial infection, is mainly caused by *Escherichia coli* and can lead to urosepsis. This case illustrates that, in patients presenting acute pyelonephritis at risk of complication but without initially severe symptoms, E-health technology can contribute to early diagnosis of vital sign deterioration and treat urosepsis quicker than the standard of care.

## Data Availability

The material contained in the manuscript has not been previously published and is not being concurrently submitted elsewhere. All data analyzed during this study are included in this case presentation. For more details, please contact the corresponding author.

## Abbreviations

GCS: Glasgow Coma Score
ICU: Intensive care unit
HR: Heart rate
MAP: Mean arterial pressure
RR: Respiration rate
SOFA: Sequential [Sepsis-related] Organ Failure Assessment
